# Identification and functional characteristics of a novel splice site variant in *L1CAM* caused X-linked hydrocephalus

**DOI:** 10.3389/fgene.2025.1588709

**Published:** 2025-05-30

**Authors:** Shijie Zhou, Hao Zhang, Xue Li, Quan Chen, Zhihong Xu

**Affiliations:** ^1^ Department of Reproductive Medicine Center, Deyang People’s Hospital, Deyang, Sichuan, China; ^2^ Deyang Key Laboratory of Birth Defects Prevention and Control, Deyang People’s Hospital, Deyang, Sichuan, China; ^3^ Department of Obstetrics and Gynecology, Deyang People’s Hospital, Deyang, Sichuan, China

**Keywords:** *L1CAM*, X-linked hydrocephalus, splice site variant, prenatal diagnostic, minigene

## Abstract

**Background:**

The *L1CAM* gene encodes an axonal glycoprotein belonging to the immunoglobulin supergene family that plays a crucial role in nervous system development. In this study, we reported a novel disease-causing mutation in the 3′ splice site of *L1CAM* and provided some insight into fetal X-linked hydrocephalus.

**Methods:**

We obtained ultrasound images and collected samples from a couple and their fetuses. Fetal samples were acquired through amniocentesis, followed by extraction of genomic DNA. We conducted copy number variation sequencing (CNV-seq), karyotype analysis, and whole-exome sequencing The mutation site of the *L1CAM* gene was verified using PCR and Sanger sequencing, with splicing effects analyzed by bioinformatics analysis via BDGP, MaxEntScan and SpliceAI, as well as *in vitro* research via minigene assays.

**Results:**

The variant c.1380-1G > A in the first male fetus, located in the intron11 3′ splice site of *L1CAM* (chrX:153868728), led to malfunction and hydrocephalus by aberrant mRNA splicing. The ultrasound examination of the fetus revealed the presence of hydrocephalus and partial agenesis of corpus callosum. In the subsequent pregnancy, the second male fetus exhibited no mutation in *L1CAM* as identified by Sanger sequencing, and the ultrasound results were within normal limits. No significant findings were observed in their CNV-seq and karyotype analysis. The second fetus was delivered uneventfully and no report of hydrocephalus through the telephone follow-up for 12 months.

**Conclusion:**

This study identified the variant c.1380-1G > A in *L1CAM* as new pathogenic mutation for the first time according to ACMG/AMP (American College of Medical Genetics and Genomics and the Association for Molecular Pathology)-based guidelines, which caused severe fetal X-linked hydrocephalus and partial agenesis of corpus callosum. This discovery expands the mutational landscape of *L1CAM*-associated disorders, highlights the diagnostic utility of integrating WES into prenatal workflows for unresolved fetal anomalies, and provides actionable insights for genetic counseling in families at risk of X-linked hydrocephalus.

## Introduction

X-linked recessive hydrocephalus constitutes the predominant congenital form of hereditary hydrocephalus among congenital anomalies, with epidemiological studies estimating its prevalence at ∼4.65 cases per 10,000 live births (Zhou et al., 2022). In prenatal diagnosis, when a fetus with hydrocephalus or other ultrasound anomalies fails to obtain a definitive diagnosis through CNV and karyotype analysis, ACMG recommends WES as the further genomic testing to yield more diagnostic information of the cases (Monaghan et al., 2020). Clinical data reveal a significant gender disparity, where 2%–15% of male neonates with congenital hydrocephalus exhibit this X-linked subtype (Kenwrick et al., 1996). Molecular pathogenesis studies have identified deleterious mutations in *L1 cell adhesion molecule* (*L1CAM*) gene as the primary etiological factor underlying X-linked neurodevelopmental disorders (Zhou et al., 2022; Bousquet et al., 2021).

The *L1CAM* gene, mapped to chromosomal region Xq28, spans 29 exons with its initial exon functioning as a noncoding regulatory element (Vos et al., 2010). The encoded L1CAM glycoprotein (1,257amino acids) represents a key component of the immunoglobulin superfamily, characterized by its transmembrane structure and cell adhesion properties (Moos et al., 1988). Functionally, this molecule plays a pivotal role in neurodevelopment through multiple interaction modalities: homophilic binding with adjacent L1CAM molecules, heterophilic associations with other Ig family members, and integrin-mediated adhesion via both RGD-dependent and -independent pathways (Linneberg et al., 2019).

Pre-mRNA splicing constitutes a critical regulatory mechanism in gene expression, wherein sequence variations frequently induce aberrant transcript formation (Scotti and Swanson, 2016). Notably, splicing dysregulation in *L1CAM* underlies the phenotypic spectrum of L1 syndrome - encompassing X-linked hydrocephalus with aqueductal stenosis (HSAS, OMIM #307000), MASA syndrome (OMIM #303350), SPG1, and corpus callosum agenesis (OMIM #304100) (Kenwrick et al., 1996; Vos et al., 2010). The clinical heterogeneity of these disorders correlates with varying degrees of molecular dysfunction (Gao et al., 2022). The splicing machinery involves conserved sequence elements (5'/3′splice sites, polypyrimidine tracts, branchpoints) and regulatory motifs (splicing enhancers/silencers), with structural RNA features further modulating spliceosome assembly (Baralle and Baralle, 2005). NM_000425.5: c.1379 + 5G>A, a mutation near the 5′splice site of intron 11, has been linked to aberrant splicing and hydrocephalus (Du et al., 1998). In contrast, mutations at the 3′splice site of intron 11 remain unreported. Pathogenic 3′splice site variants may induce diverse splicing errors including exon skipping, intron retention, pseudoexon incorporation, and cryptic splice site activation (Vaz-Drago et al., 2017).

While direct RNA analysis from affected neural tissue remains the gold standard for splicing variant characterization, practical limitations in tissue accessibility often necessitate alternative approaches (Baralle and Baralle, 2005; Whiley et al., 2014). Computational predictive tools offer preliminary insights into splicing impacts, though their clinical application requires rigorous experimental validation through functional assays (Tosi et al., 2010).

Current ClinVar database document 84 pathogenic and 62 likely pathogenic *L1CAM* variants (last accessed 30 April 2025), exhibiting a broad spectrum of molecular alterations: 37 of them are missense, 45 of them are nonsense, and 34 of them are splice site variants. Our study documents a novel splice site variant (NM_000425.5:c.1380-1G>A) identified through whole-exome sequencing, supported by bioinformatics predictions and prenatal ultrasonographic findings. Following ACMG/AMP guidelines (Jaganathan et al., 2019), we propose a provisional assessment of its pathogenicity classification based on cumulative evidence from molecular and clinical analyses.

## Material and methods

### Sample collection

The blood samples of the parents and the amniotic fluid of fetuses collected by amniocentesis were kept at −80°C.

### Bioinformatics analysis

Bioinformatics analysis was carried out to predict the splicing effect of the variant. The analysis of BDGP (available at http://www.fruitfly.org, score cut off 0.4) and MaxEntScan (available at http://hollywood.mit.edu/burgelab/maxent/Xmaxent.html) was done to determine the potential effects of variation on classic 3′acceptor consensus sites and to predict the generation of new sites (Varadi et al., 2024; Yeo and Burge, 2004). SpliceAI (https://spliceailookup. Broadinstitute.org/, accessed on 21 February 2022) was also used to predict possible splicing outcomes (Abramson et al., 2024). The three-dimensional (3D) structural models of the proteins were obtained from AlphaFold Protein Structure Database (available at https://alphafold.com/) (Zhang et al., 2023) and AlphaFold 3 (Wang et al., 2010), displayed with PyMOL Molecular Graphics System.

### CNV-Seq and karyotype analysis

CNV-Seq was performed as previously reported (Zhang et al., 2021). In brief, the workflow of CNV-seq included extracting genomic DNA, constructing a library, quality control, pooling, sequencing, bioinformatics analysis and interpreting the results. Karyotype harvest and analysis were conducted following previously established protocols (Zhang et al., 2021). Amniotic fluid was cultured in 3 mL of BIO-AMF-3 complete medium (Biological Industries, Cromwell, United States) and incubated at 37°C in a Thermo 3111 CO2 incubator (Thermo Fisher). Subsequently, amniocytes were harvested for G banding after culturing for 9–14 days. For each sample, 20 metaphase images were captured and counted using a Zeiss automatic karyotyping scanning system (Carl Zeiss, Jena, Germany). Karyotypes were described according to ISCN 2024 guidelines.

### WES

1 μg genomic DNA was extracted from 200 μL peripheral blood, using a Qiagen DNA Blood Midi/Mini kit (Qiagen GmbH, Hilden, Germany) following the manufacturer’s protocol. 50ng DNA was interrupted to 200bp around by fragmentation enzymes. The DNA fragments were then end repaired, and the 3′end was added 1 A base. Secondly, the DNA fragments were ligated with barcoded sequencing adaptors, and fragments about 320bp were collected by XP beads. After PCR amplification, the DNA fragments were hybridized and captured by WES according to the manufacturer’s Protocol. The hybrid products were eluted and collected, and then subjected to PCR amplification and the purification. Next, the libraries were quantified by qPCR. Finally, Novaseq6000 platform (Illumina, San Diego, United States), with 150 bp pair-end sequencing mode, was used for sequencing the genomic DNA of the family. Raw image files were processed using CASAVA v1.82 for base calling and generating raw data. Sequencing depth ≥30×, target coverage >95%, and Sanger sequencing validation of *L1CAM* variant with 100% concordance.

The sequencing reads were aligned to the human reference genome (hg38/GRCh38) using Burrows–Wheeler Aligner tool and PCR duplicates were removed by using Picard v1.57 (http://picard.sourceforge.net/). Verita Trekker^®^ Variants Detection System by Berry Genomics and the third-party software GATK (https://software.broadinstitute.org/gatk/) were employed for *L1CAM* variant calling. Variant annotation and interpretation were conducted by ANNOVAR (Du et al., 1998) and the Enliven^®^ Variants Annotation Interpretation System.

### Minigene assays

Minigene assay was preformed as previously reported (Coucke et al., 1994). Genomic DNA of the fetus and the mother were used to amplify the minigene regions spanning exon 11–12 and intron 12 of the *L1CAM* gene respectively, employing primers with EcoRI restriction sites (Figure 4a).The amplified products were cloned into the pSPL3 exon trapping vector by using ClonExpress II One Step Cloning Kit (Vazyme, Nanjing, China). The wild-type plasmid and the mutant plasmid were validated respectively by Sanger sequencing. The selected plasmids were prepared for further transfection. HEK293T cells were cultivated in DMEM medium containing 10% fetal bovine serum, penicillin (100 U/L), and streptomycin (100 mg/L) at 37°C in a 5% CO_2_, respectively. HEK293T cells were transfected with recombinant plasmids using Lipofectamine 2000 (Invitrogen) as per the manufacturer’s instructions. Total RNA was extracted from cells cultured for 48 h with TRIzol reagent (Cowin Biotech Co.). Reverse transcription-polymerase chain reaction (RT-PCR) was conducted with a forward primer SD6 (F: 5′‐TCT​GAG​TCA​CCT​GGA​CAA​CC‐3′) and a reverse primer SA2 (R: 5′‐ ATC​TCA​GTG​GTA​TTT​GTG​AGC‐3′). PCR fragments were evaluated by agarose gel electrophoresis, and isoforms were identified through Sanger sequencing. Assays performed in triplicate with wild-type and mutant.

## Results

### Clinical features and identification of a novel *L1CAM* mutation in a prenatal case

**FIGURE 1:**
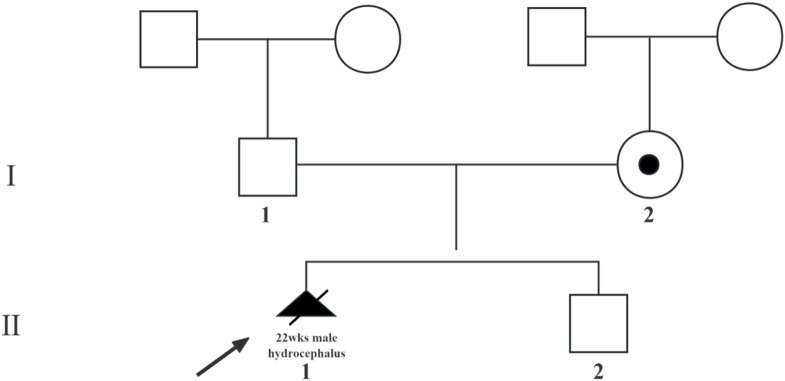
Pedigree of the X-linked hydrocephalus family. Circles denote female family members, squares denote male family members, triangles with slash denote aborted fetuses, blank symbols denates people without X-linked hydrocephalus, solid symbol subjects with X-linked hydrocephalus, core symbol denote carrier with X-linked hydrocephalus. The arrow indicates the proband.

A 27-year-old nulliparous woman was referred to our prenatal diagnosis unit at 17 weeks and 3 days of gestation due to the detection of fetal hydrocephalus via ultrasound (Figure 1). Comprehensive medical and family history assessments did not reveal any significant findings. To investigate the underlying genetic etiology, amniocentesis was performed, and amniotic fluid samples were subjected to copy number variation sequencing (CNV-seq) and G-banded karyotyping. CNV-seq and karyotyping results were prioritized to exclude common aneuploidies such as trisomy 13/18/21, and large structural rearrangements, focusing subsequent analyses on single-gene disorders.

**FIGURE 2:**
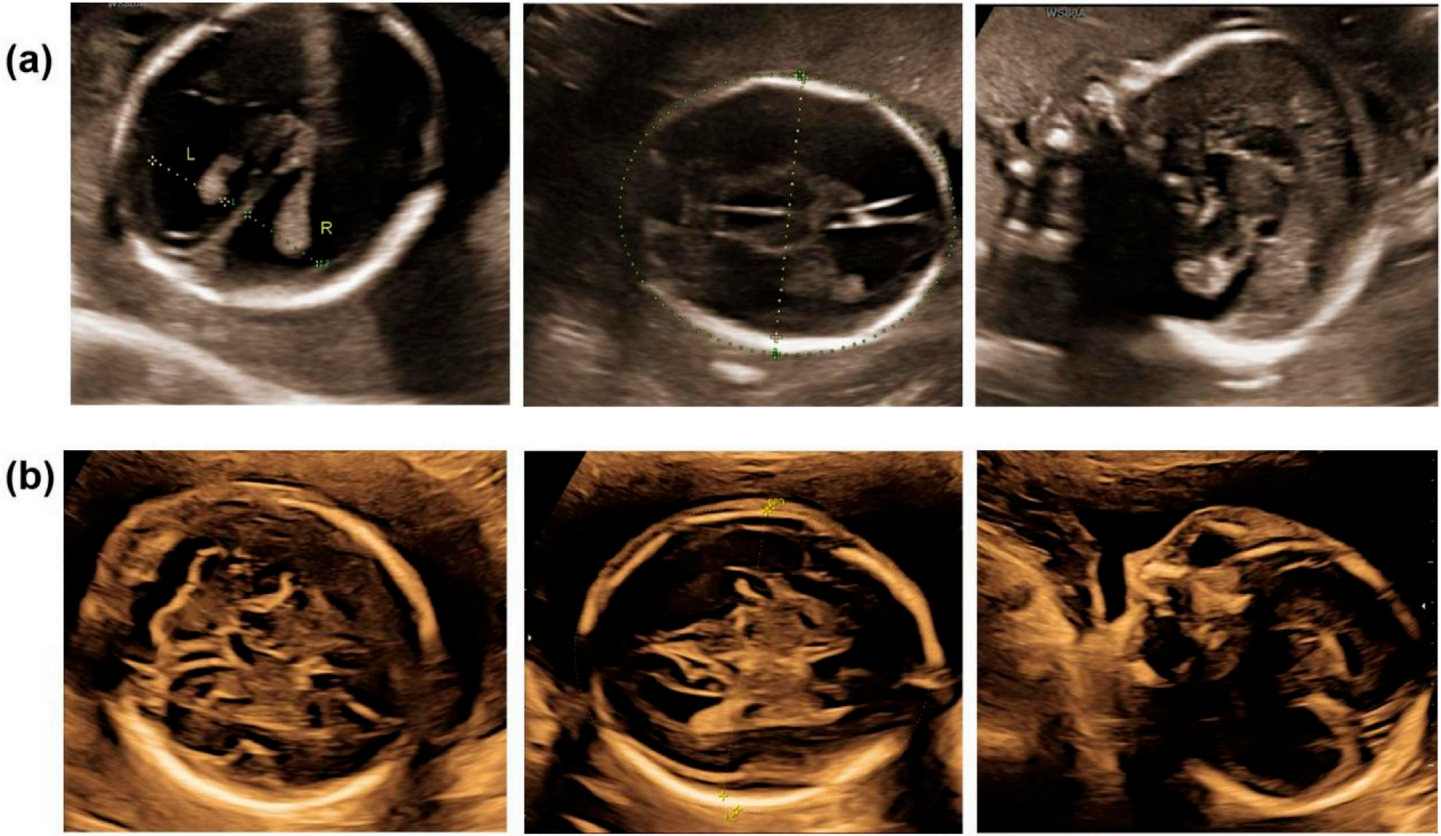
The ultrasound scan. **(a)** The ultrasound scan of hydrocephalus fetus (Ⅲ.1) in 21^+2^ weeks of gestation; **(b)** the ultrasound scan of normal fetus (Ⅲ.2) in 23^+2^ weeks of gestation.

Both analyses yielded normal results (Supplementary Figure S1a,b). However, follow-up ultrasound at 21 weeks and 2 days of gestation revealed progressive hydrocephalus with lateral ventricles measuring 15 mm and partial agenesis of the corpus callosum (Figure 2a). Given the severe and irreversible nature of these findings, the couple elected to terminate the pregnancy at 22 weeks of gestation.

**FIGURE 3:**
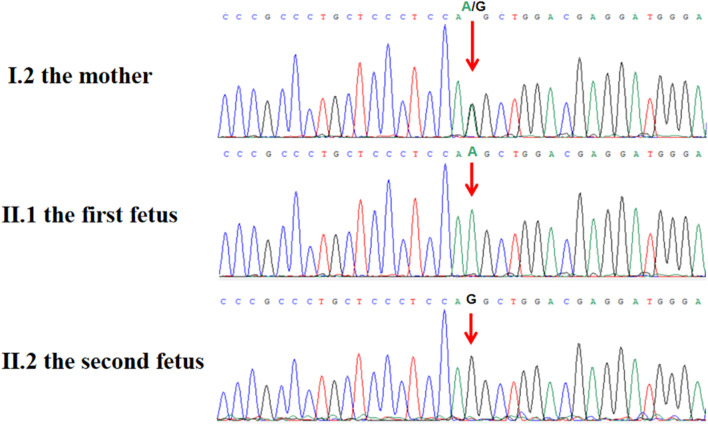
Sanger sequence chromatogram of *L1CAM* gene. Sanger sequencing showed that c.1380-1G > A was heterozygous in the mother (Ⅰ.2) and was semi-homozygous in the hydrocephalus fetus (Ⅱ.1, the proband), and was not detected in the normal fetus (Ⅱ.2).

Post-termination genetic analysis included whole-exome sequencing (WES) on both maternal and fetal DNA samples. This analysis identified a novel hemizygous splice site mutation in intron 11 of *L1CAM*, specifically NM_000425.5:c.1380-1G>A. Sanger sequencing confirmed the maternal heterozygous carrier status and the fetal hemizygous mutation (Figure 3). This variant has not been previously reported in population databases, including the 1000 Genomes Project, ExAC, EVS, or gnomAD (last accessed 26 April 2025).

In their subsequent pregnancy, the couple opted for invasive genetic testing during the second trimester. Amniocentesis at 18 weeks and 6 days of gestation revealed a normal male karyotype, and both Sanger sequencing and CNV-seq confirmed the absence of the *L1CAM* c.1380-1G>A mutation (Supplementary Figure S1c,d). Ultrasound monitoring throughout the pregnancy did not detect any structural abnormalities (Figure 2b), and the male infant was delivered at term via spontaneous vaginal delivery. At 1-year follow-up, the child exhibited normal development without signs of hydrocephalus.

### Functional analysis of the *L1CAM* splice site mutation

**FIGURE 4:**
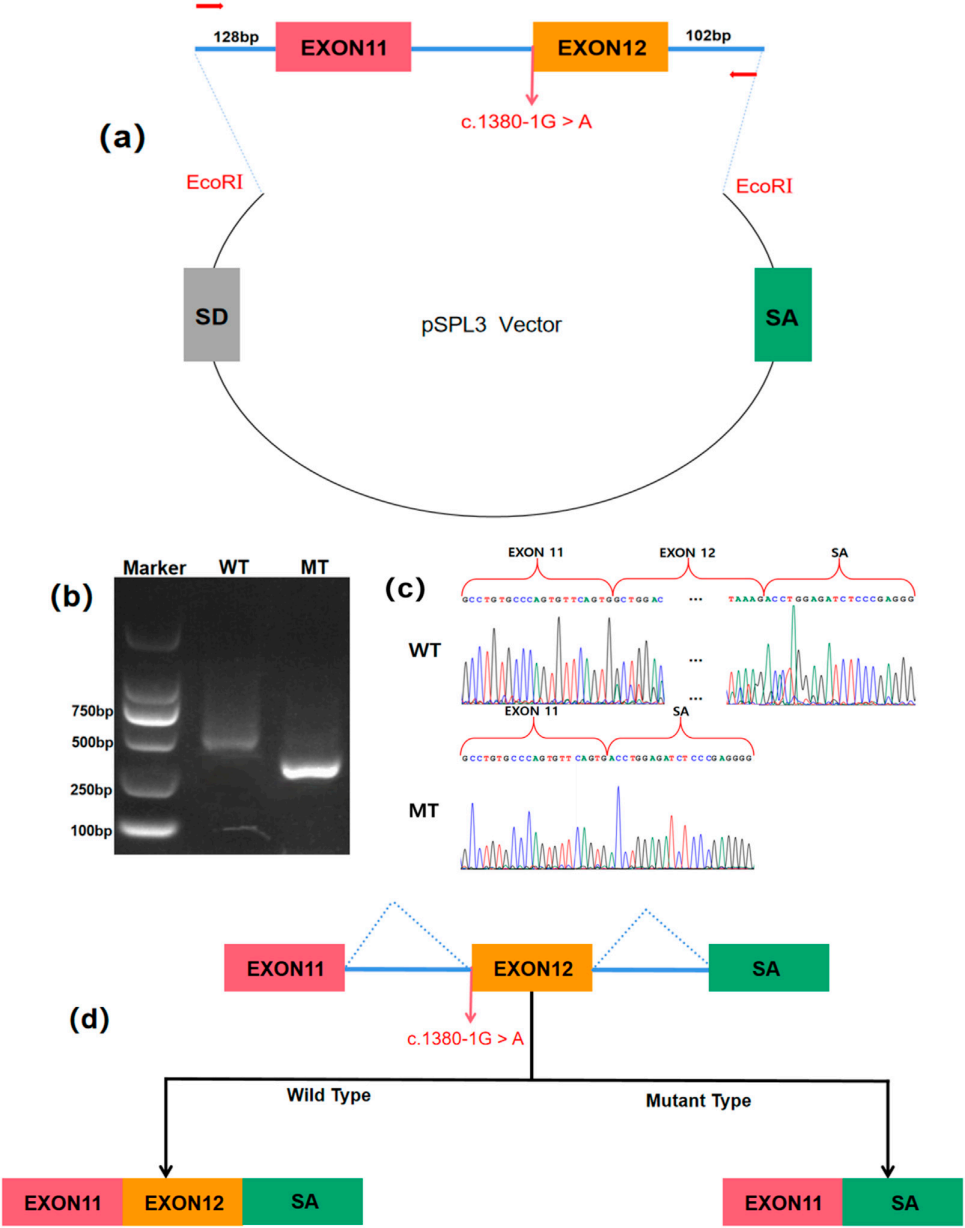
Minigene assay for *L1CAM* c.1380-1G > A mutation and schematic diagram of the splicing pattern. **(a)** The construction of a Minigene vector. **(b)** Results from gel electrophoresis of RT-PCR demonstrated the presence of bands for wild-type and mutant-type. The agarose gel electrophoresis results showed that the PCR product of pSPL3-WT exhibited a band at 546 bp, whereas pSPL3-MT displayed a band at 379 bp. **(c)** Analysis of the minigene product through sequencing. The wild-type minigene formed a normal mRNA, but the c.1380-1G > A substitution of *L1CAM* caused a splicing abnormality, which eliminated the Intron 11 canonical splice site, leading to lack of the Exon12. **(d)** The schematic diagram showed the splicing pattern of wild-type and mutant-type.

To elucidate the functional consequences of the c.1380-1G>A mutation, a minigene assay was employed. This involved constructing a pSPL3 vector containing both wild-type (WT) and mutant (MT) *L1CAM* DNA fragments (Figure 4a). Following transfection into HEK 293T cells, RT-PCR and agarose gel electrophoresis demonstrated a significant reduction in the size of the PCR product from the MT construct, from 546 bp in WT to 379 bp in MT (Figure 4b). Sanger sequencing confirmed that this size difference was due to the complete skipping of exon 12 in the MT mRNA, resulting in a 167 bp deletion (Figure 4c). These findings indicate that the mutation disrupts normal RNA splicing, leading to the exclusion of exon 12 from the mature mRNA (Figure 4d).

### Bioinformatic analysis and pathogenicity classification

**FIGURE 5:**
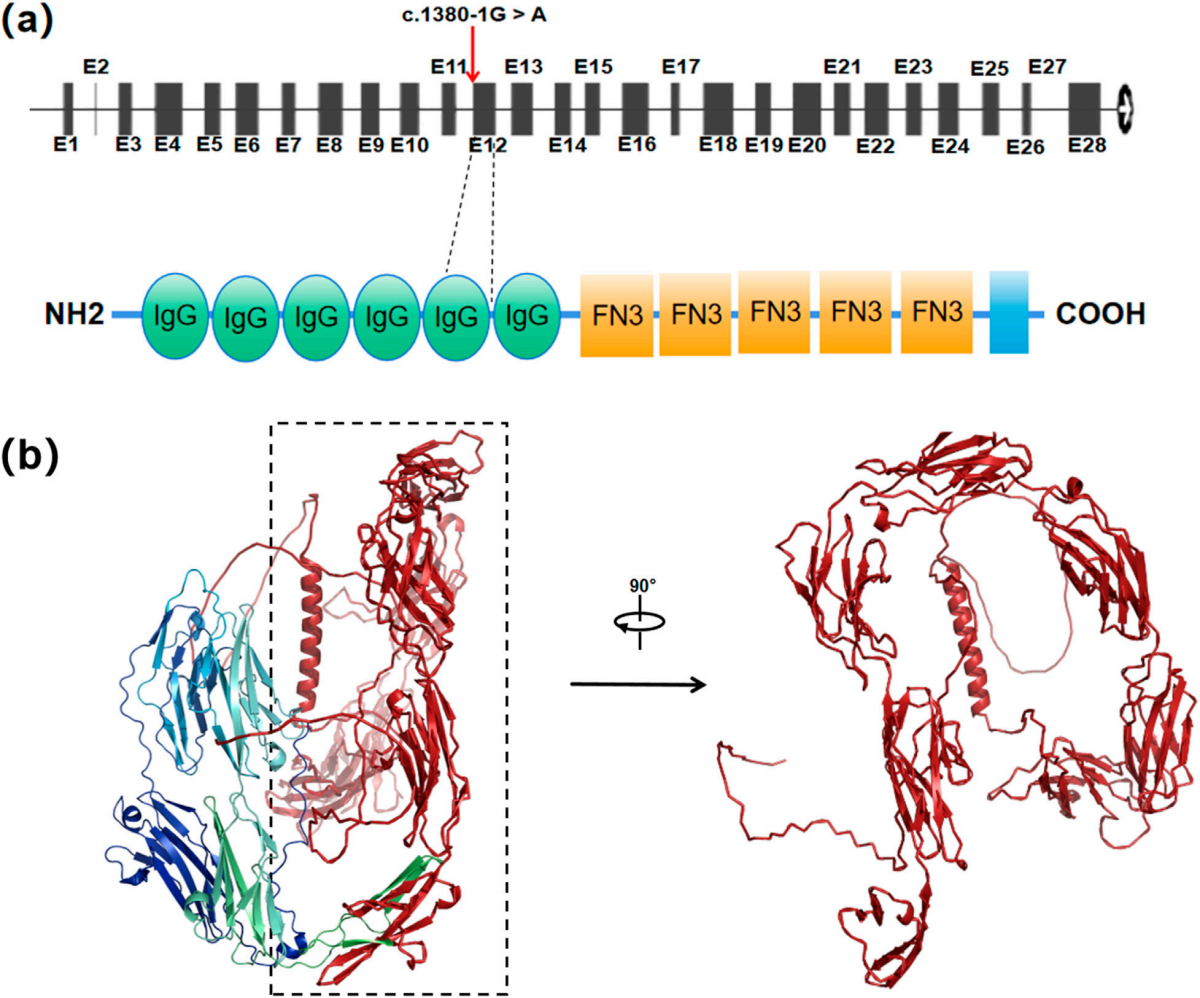
The *L1CAM* c.1380-1G > A mutation resulting in abnormal protein structure. **(a)** Locations of c.1380-1G > A in the *L1CAM* gene and protein structure. Red arrows indicate the positions of the mutation. **(b)** Predicted wild-type L1CAM protein. Red region represents a truncated protein from Ig5 domain to the end (p.Trp460Ter).

Bioinformatic analysis with BDGP and MaxEntScan demonstrated that splice site variant c.1380-1G>A reduces the score of the WT 3′ splice site from 0.94 to NA, 9.42 to 0.67 and, respectively (Table 1). And SpliceAI indicated that there is a 99% probability of acceptor loss and a 93% probability of a new acceptor gain on 1 nt downstream (Table 1). Moreover, MES predicted the same acceptor gain that shift 1 nt downstream, which might lead to p.Trp460CysfsTer51, with the score rising from −2.67 to 5.28 (Table 1). A predicted new splice site would lead to p.Trp460CysfsTer51, a frameshift mutation and truncation of the immunoglobulin (Ig) domain 5, which is crucial for its adhesive and signaling functions (Figure 5a).

**TABLE 1:** Bioinformatics analysis of *L1CAM* variant.

*L1CAM* variant	Intron/Exon	BDGP	Max ENT score analysis						SpliceAI Analysis^4^
			MES wt	MES mut	MES score change^1^	MES *de novo* SS-wt^2^	MES *de novo* SS-mut^2^	MES score change^1,3^	
c.1380-1G>A	IVS11/EX12	3′AS:0.94→NA	9.42	0.67	−92.89%	−2.67	5.28	297.75% 1-nt downstream	AL:0.99 AG:0.93→1-nt downstream

^a^ MES score changes (∆%), wildtype (wt) vs. mutant (mut).∆% > 40% score reduction are shown.

^b^ *De novo*: predicted creation of new alternative splice sites (MES≥3).

^c^ When the use of a non-canonical splice site (other than the classical GT-AG) is predicted, it is indicated in parentheses.

^d^ SpliceAI parameters were as follows: a)genome version → hg38; b)score type → raw; c)max distance → 10,000 nt; d)Illumina’s pre-computed scores → yes. Scores (≥0.2) and positions of acceptor loss (AL) and acceptor gain (AG) are shown.

The splicing pattern of the minigene indicated that splice site variant c.1380-1G>A induces exon 12 skipping, which possibly result in p.Trp460Ter, a truncation from Ig5 domain of L1CAM. Three-dimensional protein structure prediction by Alphafold3 further demonstrated the structural disruption caused by a truncated protein from the Ig domain 5, disrupting ‘FnIII-like repeats’ conformation critical for structural stability of protein extracellular regions and interactions with other partners, which the absent region highlighted in red (Figure 5b). These data demonstrate that the variant c.1380-1G>A induces splicing disorders, causing a nonfunctional L1CAM protein due to a frameshift mutation from the Ig5 domain (p.Trp460CysfsTer51) or a truncation from the Ig5 domain (p.Trp460Ter).

Based on the American College of Medical Genetics and Genomics (ACMG) and Association for Molecular Pathology (AMP) guidelines, the variant was classified as pathogenic (PVS1 + PM2 + PS3 + PP4). The PVS1 criterion was met due to the mutation affecting a canonical splice site, PM2 due to the absence of the variant in population databases, PS3 due to *in vitro* validation of minigene assay, and PP4 due to fetus’s phenotype and family history is highly specific for X-linked hydrocephalus with *L1CAM* genetic etiology.

## Discussion

This case report underscores the pivotal role of whole-exome sequencing (WES) and detailed molecular analysis in identifying rare genetic variants associated with prenatal hydrocephalus. The identification of a novel *L1CAM* splice site mutation, NM_000425.5: c.1380-1G>A, not only contributes to the understanding of the molecular mechanisms underlying X-linked hydrocephalus but also highlights the importance of comprehensive genetic testing in prenatal care. The mutation was identified in a heterozygous state in the mother and hemizygous in the aborted male fetus, leading to marked dilation of the cerebral ventricles and partial agenesis of the corpus callosum, which are characteristic features of *L1CAM*-related disorders.

Bioinformatics analysis predicted that this mutation impairs the acceptor site at the 3′splice site of intron 11, leading to splicing disorders. Although the results of the bioinformatics analysis were not completely consistent with those of the minigene experiment, both approaches indicated that the mutation causes exon 12 skipping, resulting in a frameshift truncation of the Ig5 domain (p.Trp460ProfsTer742) or a truncation from the Ig5 domain (p.Trp460Ter). This mutation is expected to disrupt the structure and function of the L1CAM protein, particularly the Ig5 domain, which is crucial for its adhesive and signaling functions.

The *L1CAM* gene encodes a transmembrane glycoprotein that belongs to the immunoglobulin superfamily and plays a critical role in neuronal migration, axon growth, and synaptic formation. The extracellular region of L1CAM is composed of six Ig domains and five fibronectin type III domains, which form a functional “horseshoe” structure essential for its activity. Deletion or truncation of the Ig5 domain, as seen in this case, has been associated with severe clinical manifestations, including hydrocephalus and corpus callosum agenesis. Consistent with these findings, previous reports have demonstrated that mutations affecting the Ig5 domain result in L1 syndrome, characterized by intellectual disability, adducted thumbs, and spastic paraplegia.

Although this specific variant has not been previously reported, similar splice site mutations in intron 11, such as NM_000425.5: c.1379 + 5G>A, have been shown to cause abnormal splicing and result in hydrocephalus (Vos and Hofstra, 2010). Other *L1CAM* splice site mutations, including c.1267 + 1G>A and c.1704-75G>T, have also been classified as pathogenic (Vos et al., 2010; Vos and Hofstra, 2010; Haspel and Grumet, 2003; Jacob et al., 2002). Splice site mutations in *L1CAM* account for over 16% of reported pathogenic variants and are known to cause exon skipping, activation of cryptic splice sites, or intron retention (Haspel and Grumet, 2003; Jacob et al., 2002). The pathogenicity of this variant was further supported by ACMG/AMP guidelines, which classified it as pathogenic (PVS1+PS3+PM2+PP1+PP3) (Jaganathan et al., 2019).

The extracellular region of L1CAM is comprised of 6 Ig domains and 5 FNⅢ domains of 11 tandem immunoglobulin-like folds, which is effective at driving homophilic and heterophilic protein-protein interactions (Adle-Biassette et al., 2013). They can fold into the region shaped as a horseshoe, which is a functional unit of the protein that is equipotent with the full-length extracellular region *in vitro* (Tapanes-Castillo et al., 2010). A report has shown that an individual with NM_000425.5: c.1380G > C transversion leading to a p.Trp460Cys substitution was diagnosed with adducted thumbs, hydrocephalus, and dysmorphic corpus callosum (Chataigner et al., 2022). Besides, a 40th week of gestation male fetus who carried the nonsense variant (c.1380G > A; p.Trp460X) result in truncation of Ig5 domain, performed pyramid hypoplasia, hydrocephalus and complete corpus callosum agenesis (Chataigner et al., 2022). The L1-6D mutant mice, which are homozygous for a deletion that removes the Ig6 domain of L1CAM, revealed typical hydrocephalus (Mohebiany et al., 2014). Moreover, the activity of the horseshoe can be regulated by Ig5 and Ig6 stabilizing the horseshoe structure (Adle-Biassette et al., 2013). According to the previous study, Ig5-6 are critical for interaction with contactin (Hortsch, 2000), which have roles in the development and function of tissuesby controlling processes of neurite extension, axon guidance, synapse formation, myelination, and axo-glia domain assembly (Hortsch et al., 2014; Lee and Rio, 2015; Szabo et al., 2015). In conclusion, currently available evidence displays the pathogenicity of this variant which splice site variants affecting structural key amino acids would disturb protein proper function (36).

The expression level of the *L1CAM* was notably low in amniotic fluid cells (37), remaining amniotic fluid samples was insufficient to conduct effective RNA analysis for verifying splicing *in vivo*. While HEK293T cells, derived from human embryonic kidney tissue, inherently lack neural-specific splicing factors, their use in functional assays remains a common surrogate for initial validation of splice site variants. This is because they retain core splicing machinery components and can recapitulate aberrant splicing patterns observed in neurodevelopmental disorders when ectopically expressing neural genes. However, the absence of neuron-specific regulators may underestimate the complexity of tissue-dependent splicing regulation. We complemented HEK293T assays with *in silico* predictions (SpliceAI, MaxEntScan) and structural modeling, which aligned with the *in vivo* consequences of exon 12 skipping. This multimodal approach strengthens confidence that the c.1380-1G>A mutation directly drives pathogenic splicing disruptions relevant to *L1CAM*-associated hydrocephalus. Additionally, further cases are needed to provide more robust evidence for the clinical and molecular findings observed in this study.

In summary, this study identifies a novel *L1CAM* splice site mutation and provides insights into its pathogenic mechanisms. The findings emphasize the importance of WES and detailed molecular analysis in prenatal diagnosis and genetic counseling for X-linked hydrocephalus. The identification of this mutation expands the mutation spectrum of *L1CAM*-related disorders and may aid in the development of targeted therapeutic strategies for this and other *L1CAM*-associated conditions.

## Data Availability

The original contributions presented in the study are publicly available. The variants data can be found in the ClinVar database (accession number: SCV005901578).
