# Correction: Identification and functional characteristics of a novel splice site variant in *L1CAM* caused X-linked hydrocephalus

**DOI:** 10.3389/fgene.2025.1676267

**Published:** 2025-09-10

**Authors:** Shijie Zhou, Hao Zhang, Xue Li, Quan Chen, Zhihong Xu

**Affiliations:** ^1^ Department of Reproductive Medicine Center, Deyang People’s Hospital, Deyang, Sichuan, China; ^2^ Deyang Key Laboratory of Birth Defects Prevention and Control, Deyang People’s Hospital, Deyang, Sichuan, China; ^3^ Department of Obstetrics and Gynecology, Deyang People’s Hospital, Deyang, Sichuan, China

**Keywords:** *L1CAM*, X-linked hydrocephalus, splice site variant, prenatal diagnostic, minigene

In the published article, there was an error in the **Result** Section, Bioinformatic analysis and pathogenicity classification, Paragraph 3.

The corrected text appears below.

“Based on the American College of Medical Genetics and Genomics (ACMG) and Association for Molecular Pathology (AMP) guidelines, the variant was classified as pathogenic (PVS1 +PM2 + PS3 + PP4). The PVS1 criterion was met due to the mutation affecting a canonical splice site, PM2 due to the absence of the variant in population databases, PS3 due to *in vitro* validation of minigene assay, and PP4 due to fetus’s phenotype and family history is highly specific for X-linked hydrocephalus with *L1CAM* genetic etiology.”

The original article has been updated.

