# Supplementary material for: Identification and functional characteristics of a novel splice site variant in L1CAM caused X-linked hydrocephalus
Source: Front Genet. 2025 May 30;16:1588709. doi: 10.3389/fgene.2025.1588709 (PMC12162546; doi:10.3389/fgene.2025.1588709)
Supplement: Supplementary file 1 [file DataSheet1.docx]

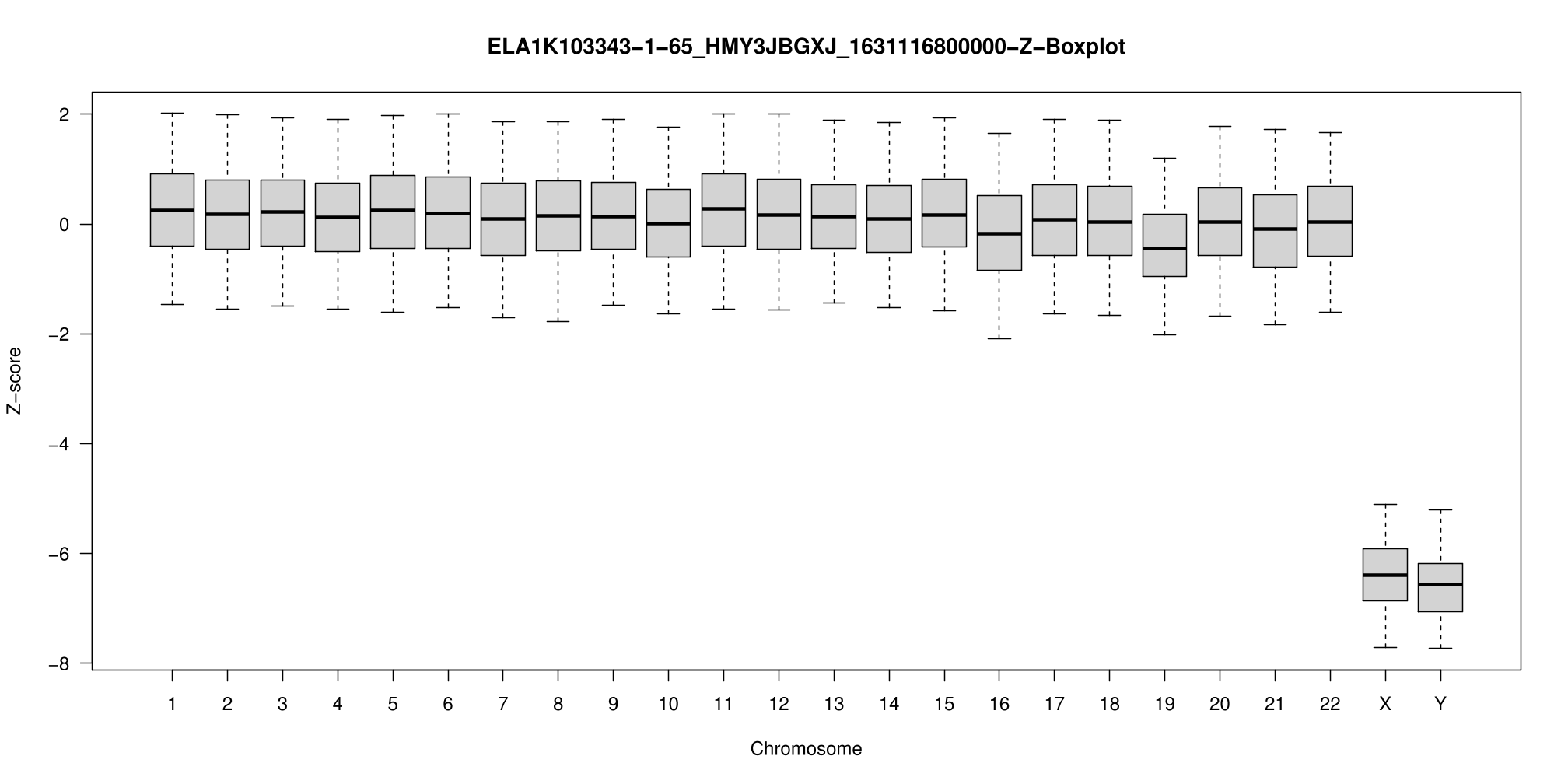

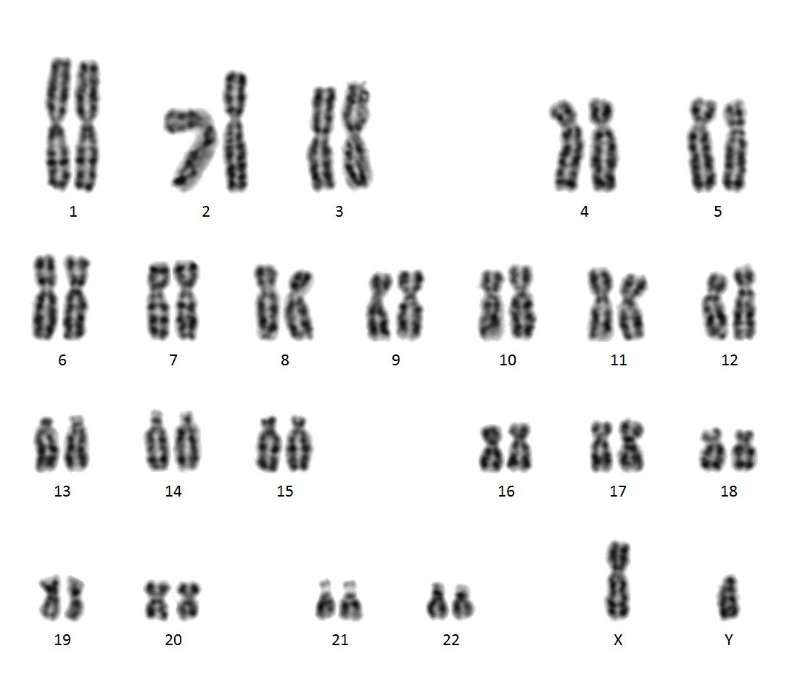


**(b)**

**(a)**


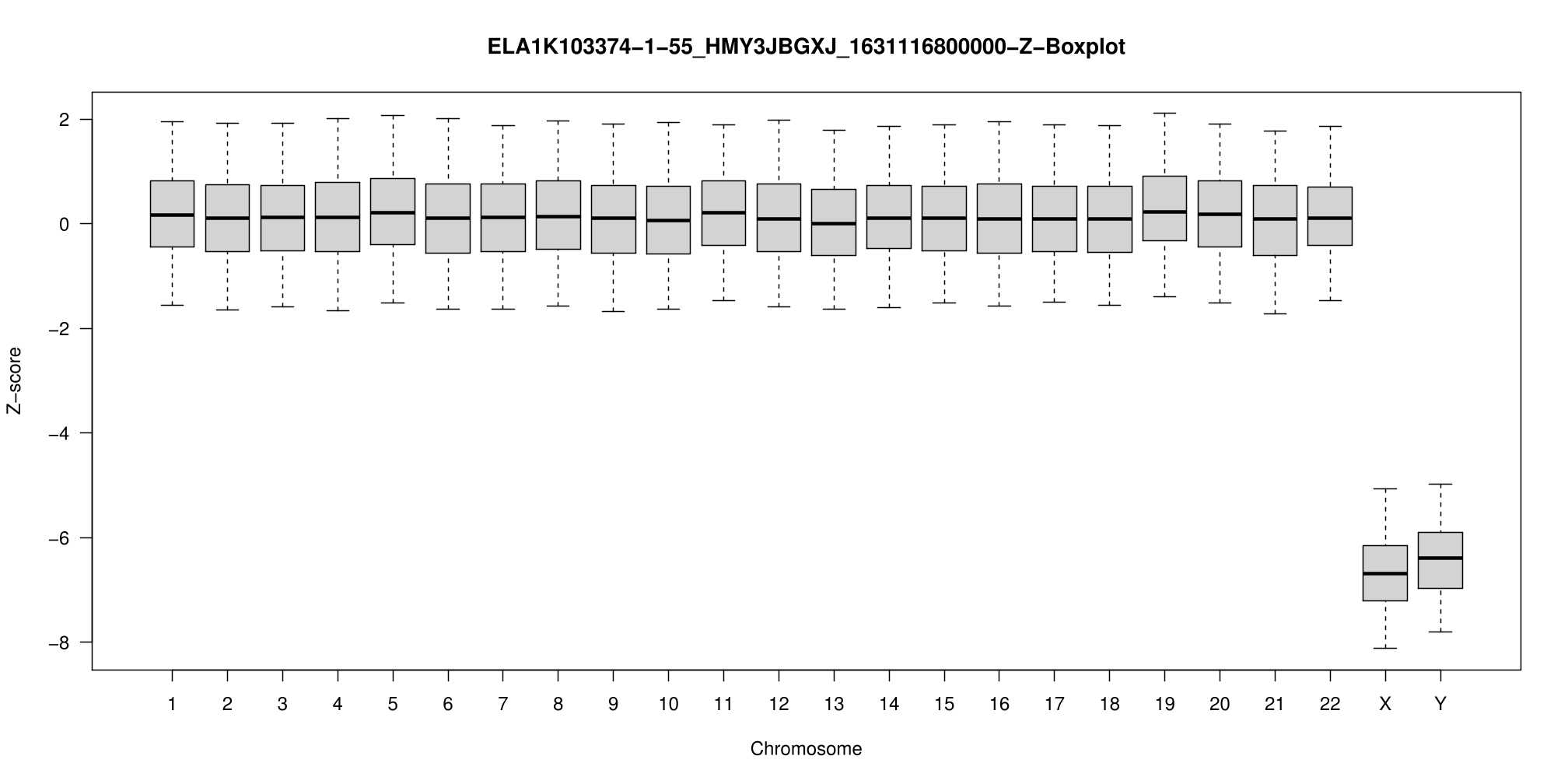


**(c)**


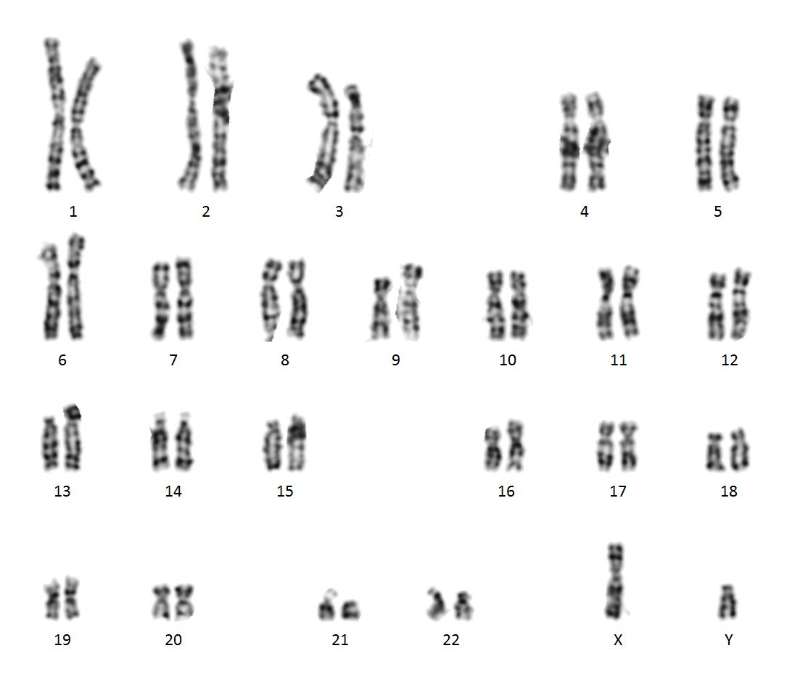


**(d)**

FIGURE S1 The CNV-Seq Z-score in each chromosome and karyotype analysis for fetuses. The gray rectangle represents the 95% z-score area, the middle black line represents the mean z-score, and the black lines at both ends of the dashed line represent the maximum/minimum z-score of the measured CNV, respectively.The number below each chromosome is the identity of that chromosome. (a) the CNV-Seq Z-score and (b) karyotype analysis results for the fetus with hydrocephalus (Ⅲ.1); (c) the CNV-Seq Z-score and (d) karyotype analysis results for the normal fetus (Ⅲ.2). Both fetuses exhibited no abnormalities in either karyotype analysis or CNV-Seq.
